# Keratoconus associated with choroidal neovascularization: a case report

**DOI:** 10.1186/1752-1947-4-58

**Published:** 2010-02-19

**Authors:** Joo Youn Oh, Hyeong Gon Yu

**Affiliations:** 1 Department of Ophthalmology, Seoul National University College of Medicine, Seoul, Korea; 2 Seoul Artificial Eye Center, Seoul National University Hospital Clinical Research Institute, Seoul, Korea

## Abstract

**Introduction:**

Keratoconus and choroidal neovascularization can occur as a result of dysfunction of the epithelium and its basement membrane.

**Case presentation:**

A 17-year-old Asian man, who was diagnosed with myopic choroidal neovascularization in both eyes and who subsequently underwent intravitreal injection of ranibizumab (Lucentis^®^) five times over six months, presented with further vision decrease and pain in his right eye. Examination showed corneal steepening and stromal edema in the inferocentral cornea of his right eye, both of which were indicative of advanced keratoconus with acute hydrops. Corneal topography also showed features consistent with keratoconus in his left eye. Fluorescein angiography and optical coherence tomography revealed choroidal neovascularization-associated subretinal hemorrhages and lacquer cracks in both eyes.

**Conclusion:**

Keratoconus and choroidal neovascularization, possibly resulting from dysfunction of the epithelium and its basement membrane, can occur together in the same individual. This would suggest a possible connection in pathogenesis between these two conditions.

## Introduction

Keratoconus is an idiopathic, progressive, noninflammatory ectasia of the lower central cornea, which possibly results from a disorder originating in the corneal epithelial basement membrane [1]. It has been associated with a variety of ocular disorders, including retinitis pigmentosa [2,3], macular coloboma [3,4], Leber's congenital amaurosis [5], retinal aplasia [4], cone-rod dystrophy [6], and central serous chorioretinopathy [7]. However, to the best of our knowledge, keratoconus has not previously been reported in association with choroidal neovascularization (CNV). CNV, which is accompanied by ruptures in Bruch's membrane, is one of the most sight-threatening complications in patients with pathologic myopia. While the clinical characteristics of keratoconus and CNV have been extensively described, the precise pathogenesis of these two diseases is unknown.

We report the unusual coexistence of bilateral keratoconus and choroidal neovascularization in a young man. The possible association between these two disorders is also discussed.

## Case presentation

**Figure 1:**
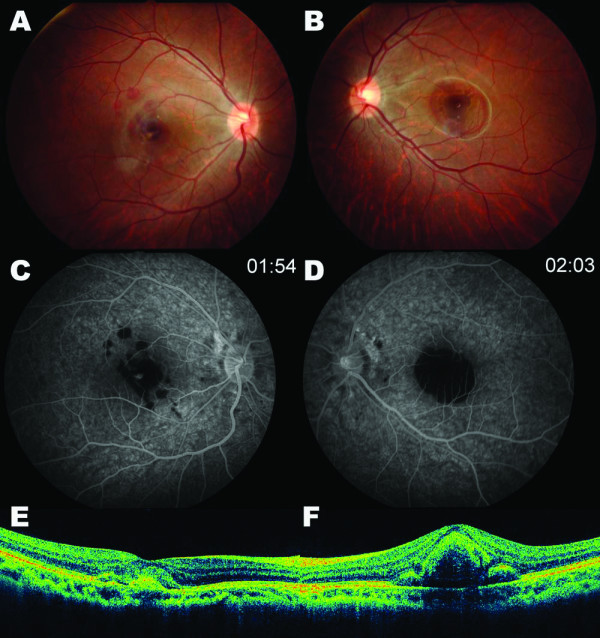
**Fundus photography (A, B), fluorescein angiography (C, D), optical coherence tomography (E, F) of both eyes**. Submacular hemorrhages are shown in both eyes (A, B). Fluorescein angiography revealed blocked fluorescence in the maculae of both eyes due to subretinal hemorrhages (C, D) with focal leakage at the level of retinal pigment epithelium in the right eye (C). Optical coherence tomography revealed multiple interruptions in Bruch's membrane with subretinal elevation in both eyes (E, F).

A 17-year-old Asian man presented with painless bilateral reduction in his vision. Vision loss had started two months previously in the right eye and two days previously in the left eye. The patient had no history of systemic disease or trauma. His best corrected visual acuities (BCVA) were 20/500 in the right eye and 20/1000 in the left eye. Refractions were -6.5 -1.5 × 180° in both eyes. Axial lengths were 29.35 mm in the right eye and 28.37 mm in the left eye. Fundus examination showed subretinal hemorrhages in the maculae of both eyes (Figure 1A, B). Fluorescein angiography (FA) revealed subretinal hemorrhages in both eyes accompanied by focal leakage at the level of the retinal pigment epithelium (RPE), which was suggestive of CNV in the right eye (Figure 1C, D). Optical coherence tomography (OCT) revealed breaks in Bruch's membrane with subretinal elevation in both eyes (Figure 1E, F).

**Figure 2:**
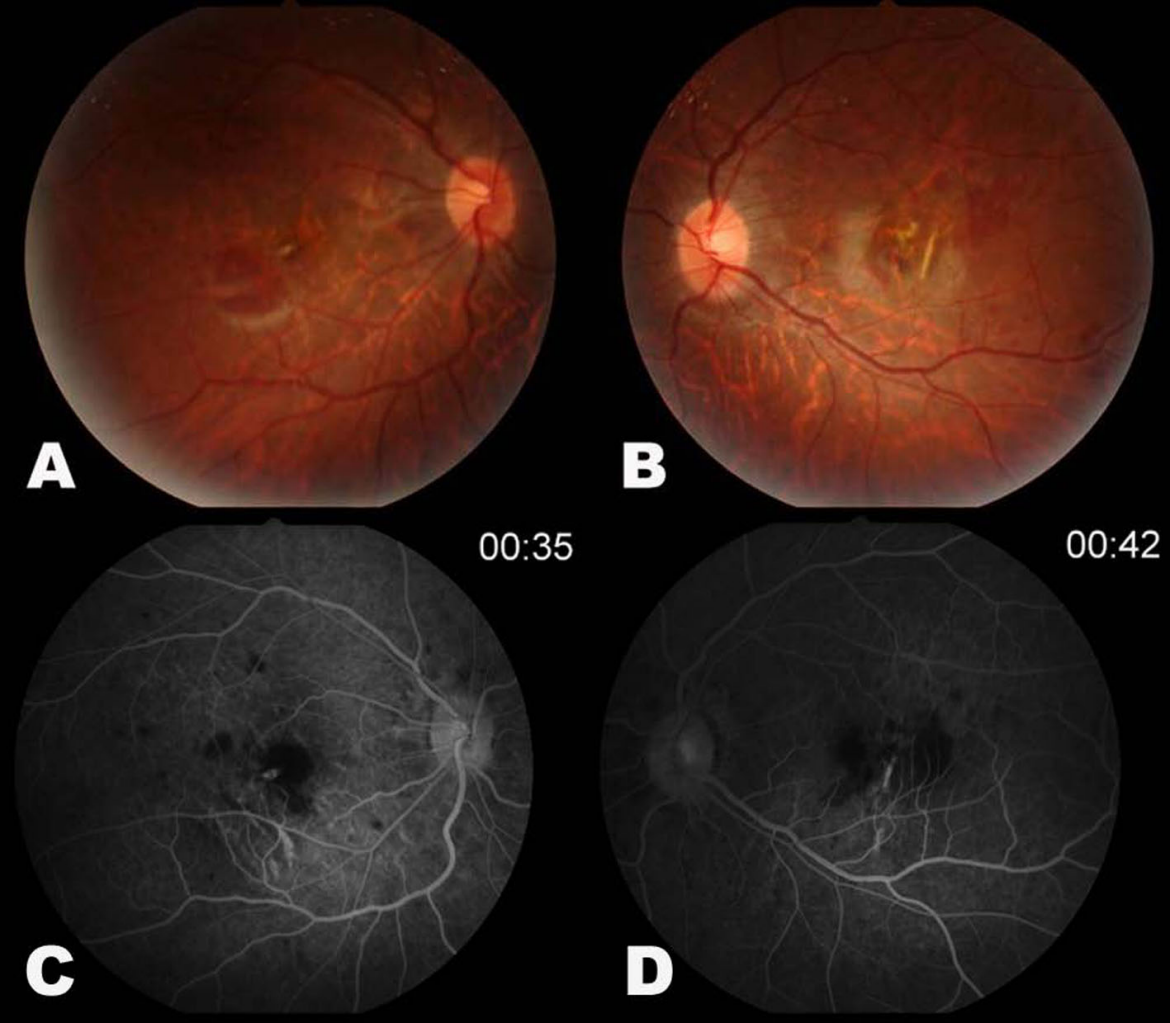
**Fundus photography (A, B) and fluorescein angiography (C, D)**. Lacquer cracks are prominent in both eyes after subretinal hemorrhages subsided.

The patient was diagnosed with CNV in both eyes, and he underwent bilateral intravitreal injections of ranibizumab (Lucentis^®^, Genetech, San Francisco, CA, USA) five times over six months. The subretinal hemorrhages decreased bilaterally, and visual acuities improved (Figure 2). After subretinal hemorrhages had subsided, lacquer cracks were clearly seen in both eyes.

**Figure 3:**
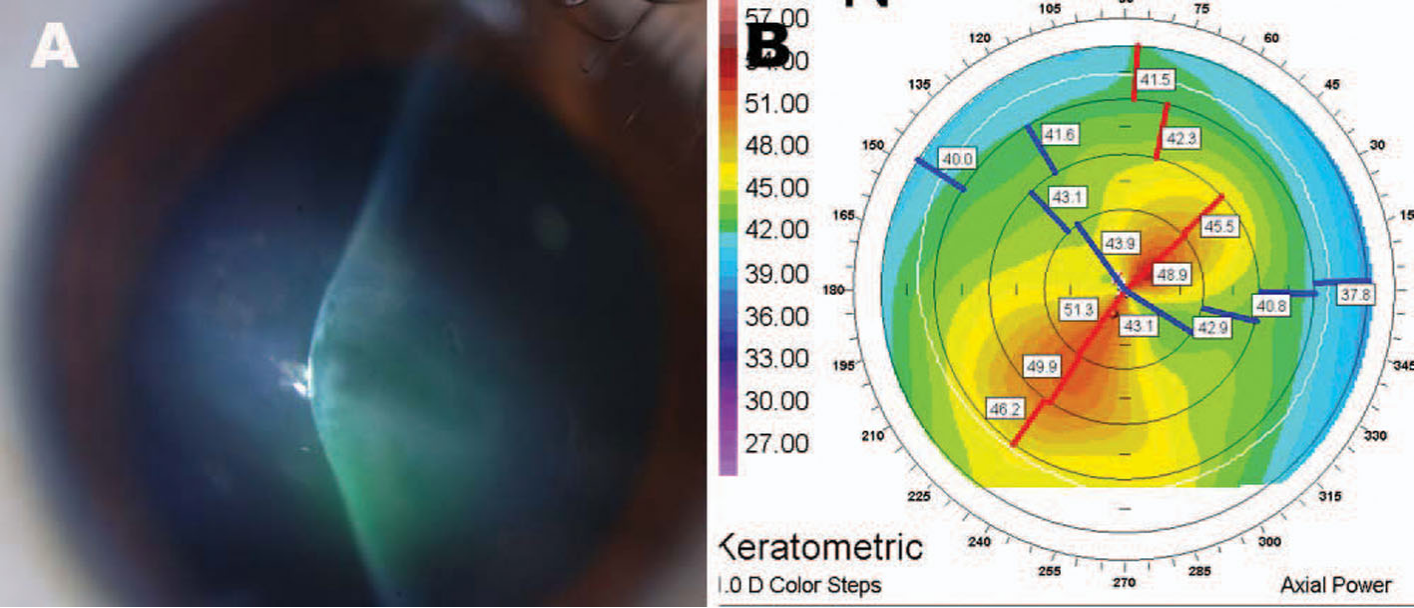
**Anterior segment photography of the right eye (A) and corneal topography of the left eye (B)**. Corneal steepening is seen in the inferior paracentral area and stromal edema with the rupture in Bowman's layer (A). Irregular astigmatism is seen with inferosuperior asymmetry and corneal steepening, indicative of keratoconus of the left eye (B).

Six months after initial presentation, the patient complained of a sudden, painful visual decrease in his right eye. On examination, his BCVA in the right eye was finger count. Slit-lamp biomicroscopy revealed corneal steepening in the inferior paracentral area and stromal edema in his right eye with rupture of Bowman's layer, signs characteristic of advanced keratoconus and acute hydrops (Figure 3A). Corneal topography of his left eye showed irregular astigmatism and inferosuperior asymmetry, both of which were indicative of keratoconus (Figure 3B).

## Discussion

Keratoconus is known to be a basement membrane disorder that alters the cell-substrate interaction between the corneal epithelium and its basement membrane [1]. The hypothesis regarding the pathogenesis of keratoconus is based on the associated histopathologic changes noted in the extracellular matrix (ECM) within the cornea: fragmentation of the epithelial basement membrane, superficial linear ruptures in Bowman's layer, and delayed development of folds in Descemet's membrane [1,8]. Moreover, corneas with keratoconus are characterized by upregulation of various enzymes related to remodeling of the ECM and downregulation of genes encoding collagen type IV, laminin, and fibronectin; these downregulated genes encode for the molecules responsible for cell attachment in various basement membranes throughout the body [9-11]. These molecules are also involved in the attachment of the RPE to Bruch's membrane [12,13]. Furthermore, alterations in the genes encoding tissue inhibitor of metalloproteinase 3 (TIMP-3) and cathepsin are also found in a cornea with keratoconus [14,15]. TIMP-3 is involved in the remodeling of the RPE and Bruch's membrane and plays an important role in the regulation of choroidal vascularization [13]. Cathepsin, which contributes to the initiation and/or progression of keratoconus, is also responsible for photoreceptor homeostasis, and deregulation of cathepsin is associated with macular degeneration [15].

The patient in our report showed lacquer cracks (breaks in Bruch's membrane), subretinal hemorrhages with CNV, and keratoconus in both eyes. Keratoconus became so severe in one eye that acute hydrops (a rupture in Bowman's layer) developed. This case may represent the unusual coexistence of separate disease entities. However, given that keratoconus and myopic CNV have the abovementioned molecular basement membrane abnormalities in common, it is possible to elucidate a correlation between these two diseases. Moreover, this patient had CNV in both eyes although he was young and not highly myopic and keratoconus progressed so rapidly as to develop acute hydrops. These rapid and severe changes in Bruch's membrane and Bowman's layer in the same patient might represent a possible association between these two disease entities.

Moreover, given the common posterior segment disorders associated with keratoconus in this and other investigators' reports, it would be wise to perform a thorough retinal examination before corneal transplantation in order to detect coexisting retinal disorders in patients with keratoconus.

## Conclusions

We noted bilateral CNV and keratoconus in a young man. Both diseases might represent abnormalities of the interaction between the epithelium and its basement membrane. Further investigation is necessary to determine the possible correlation between the two disorders and investigate the precise mechanisms behind these diseases.

## Patient's perspective

I hope my case will be helpful for other patients who suffer from a disease like mine.

## Abbreviations

BCVA: best corrected visual acuities; CNV: choroidal neovascularization; ECM: extracellular matrix; FA: fluorescein angiography; OCT: optical coherence tomography; RPE: retinal pigment epithelium; TIMP-3: tissue inhibitor of metalloproteinase 3.

## Consent

Written informed consent was obtained from the patient for publication of this case report and any accompanying images. A copy of the written consent is available for review by the Editor-in-Chief of this journal.

## Competing interests

The authors declare that they have no competing interests. This material is not under consideration for any presentation and has not been previously presented at any meeting.

## Authors' contributions

OJY analyzed and interpreted the patient data and was a major contributor in writing the manuscript. YHG interpreted the patient data and supervised the overall concept of the manuscript. Both authors read and approved the final manuscript.
